# Structured Evaluation of Rehabilitation Programs Outcomes in Psychiatry: Application of a Recovery-Centered Model

**DOI:** 10.1007/s11126-021-09884-0

**Published:** 2021-05-25

**Authors:** Simone Vanzetto, Matteo Zabotto, Federica Fasciana, Alberto Varinelli, Giovanna Cirnigliaro, Luca Ferrara, Bernardo Dell’Osso, Caterina Viganò

**Affiliations:** 1 Department of Mental Health, Department of Biomedical and Clinical Sciences Luigi Sacco, University of Milan, Milan, Italy; 2 Psychiatry Unit 2, ASST Sacco-Fatebenefratelli, Via G.B. Grassi, 74, 20157 Milan, Italy; 3 Psychiatry Unit 1, Department of Mental Health, ASST Fatebenefratelli-Sacco, Milan, Italy; 4 Department of Health Sciences, “Aldo Ravelli” Center for Neurotechnology and Brain, Milan, Italy; 5 Department of Psychiatry and Behavioral Sciences, Bipolar Disorders Clinic, Stanford University, Stanford, CA USA; 6 Centro per lo studio dei meccanismi molecolari alla base delle patologie neuro-psico-geriatriche, University of Milan, Milan, Italy

**Keywords:** Rehabilitation, Recovery, High assistance rehabilitative community, Structured outcome indicator model, Psychosocial functioning

## Abstract

Rehabilitation is oriented to psychiatric patients’ recovery through specific techniques and structured projects, not yet fully standardized, carried out in territorial services. This study aims to apply an operational structured outcome indicator model (hospitalizations, continuity of care, LAI treatment adherence, working support) through a recovery-centered model in a rehabilitation community in Milan. This observational-retrospective study included 111 patients from a University High Assistance Rehabilitation Community (C.R.A.) based in Milan. Psychopathological and psychosocial functioning was evaluated with Kennedy Axis V, Brief Psychiatric Rating Scale (BPRS), Life Skills Profile (LSP), AR module of the VADO scale. Statistical analyses were performed using SPSS software version 19. Student *t* test and Wilcoxon Test were used to analyze quantitative variables, while McNemar test for qualitative variables. The minimum level of significance was set at 0.05 (*p* <0.05). The results showed that CRA rehabilitation program led to significant improvement in global functioning in terms of hospitalization reduction; improved continuity of care; stable adherence to psychopharmacological treatment with Long Acting Injectable (LAI) antipsychotics; stable employment maintenance during the year following discharge from the CRA. This study confirmed the utility of a structured outcome indicator model and highlighted its feasibility in daily clinical context of a rehabilitative community. Our results supported the effectiveness of a community-based rehabilitation program to improve individual functioning and clinical stability. However, further studies are required to better achieve the development of a recovery-oriented rehabilitation model and rigorously define an outcomes evaluation model.

## Introduction

Psychiatric diseases are debilitating disorders and a leading cause of ill-health and disability worldwide, estimating that at least one out of four people is affected by mental illness either directly or indirectly [1]. The consequence of mental illness is the progressive individual impoverishment, which can lead to isolation, promoting chronicity of the disease. In 2011, the World Health Organization defined rehabilitation as "a set of interventions aimed at helping people who experienced disability to achieve and maintain adequate functioning in everyday life”. Psychiatric rehabilitation aims to identify, reduce and prevent the causes of disability, helping a person to develop and use their resources in order to gain more confidence and self-esteem, counteracting the risk of chronic psychiatric illness [2, 3]. According to Libermann definition, “disability is where we start, recovery is our destination, and rehabilitation is the road we travel [4]. In that sense, nowadays, the concept of Recovery is gaining more attention in literature due to the evidence that clinical remission exclusively may not represent the only therapeutic target. In fact, functional impairment, social isolation and unemployment still remain as an area needing to be focused on. Recovery is a long and complex process, person-centered, with the aim of becoming aware of one's limitations and vulnerability. The patient represents the fulcrum of this process, overcoming the concept of mental illness as an immutable and chronic disease, to re-gain an effective role in the society [5].

One of the main aims of Recovery is to address disability due to psychiatric illness that may be very severe in psychiatric disorders. Thus, Mental diseases can affect patients’ life, by worsening their daily functioning and compromising their quality of life [6]. Based on these assumptions, to treat mental disease efficiently, some authors underline the importance of extra-hospital facilities with preventive, rehabilitative and therapeutic functions that are able to address all the impaired domains and not exclusive symptoms remission [4, 5]. For these reasons psychiatric rehabilitation is a set of interventions that should be proposed by “recovery-oriented services for people with disabilities associated with longer-term mental health problems [7]. A lot of studies underline the efficacy of psychiatric rehabilitation in functional recovery through the application of medical approaches and cognitive-behavioral therapy principles into rehabilitation programs [8–11]. Regarding that, during the past several decades, psychosocial rehabilitation got more importance in individuals with long-term mental illnesses [12]. In the last years there has been an evolution of rehabilitation models towards the application of Evidence Based Medicine also in this area [13]. Evidence-based psychiatric rehabilitative interventions focused on a person-centered care plan that ensures people to receive the most appropriate services and supports over time, identifying personal goals and preferences [14]. Furthermore, in high-income countries in Europe, this change consisted in psychiatric deinstitutionalization that led to outpatient care and community-based services [15]. Even today, no countries in Europe have fully implemented the rehabilitation principles in their national mental health policies [16]. Implementing rehabilitation and community mental health care facilities should be a priority also in Italian psychiatric system, as suggested by different authors [17, 18] to reduce stigmatization and improve patients’ global functioning and autonomy in day-life activities. Another recent challenge is the gap between an increasing rehabilitation demand and economical found devoted to this intervention [17]. Hence, optimizing the rehabilitative interventions and resources by the identification of efficacy model of outcome may represent a feasible approach to this issue. Different studies show the necessity of the creation of an outcomes evaluation model in community-rehabilitation programs, evaluating psychopathological, relational and social functioning [19–22]. There are few Italian studies conducted in real clinical settings and, to our knowledge, this is the first one suggesting the evidence of recovery-based community program efficacy by evaluating outcome indicators in real world contest: number and duration of hospitalizations, continuity of care, adherence to LAI treatment, working condition and possible attachment to forms of work support.

## Methods

The present study aims to evaluate the feasibility of a rehabilitative outcome model for measuring the improvement and the maintenance over time at the end of a residential rehabilitation program, for patients with major psychiatric disorders. We propose a set of outcome indicators based also on validated scales [19–23]. Furthermore, a secondary goal is to determine the efficacy of a recovery program by restoring impaired functioning in different domains such as work, social life and clinical features such as treatment compliance, hospitalization and continuity of care with a one-year follow-up compared to 1 year-time before the rehabilitative program.

This is a retrospective observational study that involved all the resident patients in a University High Assistance Community Rehabilitation (CRA) based in Milan, between January 1, 2011 and June 1, 2017.

This Rehabilitation Community provides 24 hours/7 days healthcare assistance to patients with major psychiatric disorders such as psychotic disorders, affective disorders and severe personality disorders. All the resident patients are in charge at Psycho-Social Centers (CPS), corresponding to the Centers for Mental Health (CSMs) in the other Italian regions, a territory-based psychosocial outpatients service responsible for continuity of care. The patients carry out different residential rehabilitative programs:

- Post-Acute (RPA), 3 months-program, possibly renewable up to 6 months following an acute episode of psychiatric disease
- High Intensity (RHI), 18 month-program, extendable up to 24 months, for specific rehabilitative program.

The Post-Acute program is the only rehabilitative intervention available for patients aged >50 years old. In both these interventions, resident CRA patients may come from their own home or inpatients unit, identified as Psychiatric Diagnosis and Treatment Service (SPDC), according to psychiatric territorial services.

The first step of psychopathological and psychosocial functioning evaluation is based on the use of specific scales such as, Kennedy Axis V [24, 25], Brief Psychiatric Rating Scale (BPRS) [26], Life Skills Profile (LSP) [27, 28], AR module of the VADO scale [29].

The second step consists of the planning of individual goals within the therapeutic rehabilitation pathway. Individual and group activities, psycho-educational interventions, cognitive remedies, expressive and psychotherapeutic activities are organized to improve and develop social and relational skills in order to stimulate and facilitate the readmission into everyday life. These activities are set and selected in agreement with the community patients in order to achieve the rehabilitation goals.

In the present study we consider anamnestic, clinical, socio-demographic and therapeutic data collected from CRA’s and CPS’ medical records, and from hospital information systems by psychiatrists or trainee students. Patient data of one-year before and one-year follow-up were collected through the CPS's medical records and SISM (*Sistema Informativo per la Salute Mentale* - Mental health information system).

The evaluated data were: gender, education level, housing and work condition in the 12 months before admission, psychiatric diagnosis, organic and psychiatric comorbidities, use of substances /alcohol, age of onset, duration of illness and duration of untreated illness (DUI), number of hospitalizations lifetime, previous rehabilitation experiences.

The following descriptive parameters were also taken into consideration: RHI or RPA (used to stratify the sample), number of hospitalizations during the project, psychopharmacological therapy assumed both at the entrance and at the discharge (mono-polytherapy, depot).

We identified and evaluated in 12 months before and after the rehabilitative program the following outcome indicators according to the Italian National State-Regions Agreement of cure program care [30] and literature data: number and duration of hospitalizations [31, 32], continuity of care [33], adherence to LAI treatment [34], working condition and possible supported work services [35].

Number and duration of hospitalizations was identified as an outcome given the evidence that non acute inpatient mental health rehabilitation reduces re-hospitalization, which will have benefits for both consumers and health services [31, 32].

LAI treatments and continuity of care, according to literature, reduced relapses and hospitalizations so represents a valid variable to evaluate the rehabilitative program efficacy [19, 33, 34, 36].

Working condition and possible supported work services: several studies have shown that psychiatric patients have a high risk of losing their jobs and employment rate was lower than the general population, ranging from 10 to 25% [35, 37]. According to NICE Guidelines, one of the main aims of a rehabilitative program is to provide assistance to find a job as well as to develop work skills and employment maintenance [38–40].

Resident patients were also tested with psychometric scales, BPRS [26] for psychopathological variables, GAF [41] for global functioning. 

Test evaluation was performed at the entrance (T0), repeated at the end of the project (T1) and 12 months after discharge (T2) to assess the maintenance over time. The clinical indicators were derived from the revision of the medical records and SISM data.

### Statistical Analyses

Statistical analyses were performed with SPSS software version 19. Descriptive analyses were calculated using frequencies and percentages for categorical variables and means and standard deviations (SDs) for continuous variables. We carried out first a descriptive analysis of the total sample, which was also dichotomized in RHI and RPA subgroups. The descriptive analysis was then repeated in the above-mentioned subgroups. The differences in clinical indicators in the 12 months before and after the program were then evaluated. Student *t* test was used for paired data in case of the quantitative variables with normal distribution. The Wilcoxon test, for not independent ordinal samples, was used for quantitative variables that had a non-parametric distribution. The McNemar test was used for nominal samples that were not independent, in the case of qualitative variables. The minimum level of significance for all statistical analyses was set at 0.05 (*p* <0.05), which indicates a probability of error less than or equal to 5% and higher levels of significance at 0.01 (*p* <0.01) and 0.001 (*p* <0.001; very significant).

The present protocol was approved by the local Ethics Committee and the written informed consent was obtained from the patients or relatives after a full description of the study.

All the authors certify responsibility for the integrity of the paper's content. All the authors also declare no conflict of interest.

## Results

We evaluated 129 patients who were admitted to CRA for an overall period of 78 months (from the 1st January 2011 to the 30th June 2017). Among them, 18 were not considered in the analysis due to the premature interruption of the rehabilitation program (3 of them were hospitalized).

Then, the remaining 111 patients were included in statistical analysis. 56 (50.5%) of the sample were females and 55 (49.5%) were males with a mean age of 41.8 years (± 11.9) and a duration of illness of 18 years (± 11.1). 44 (39.6%) patients presented a diagnosis of Psychotic Disorders (Schizophrenia, Delusional Disorder, brief Psychotic Disorder and Drug-induced psychosis); 10 (9%) with Schizoaffective disorder; 34 (30.6%) with Bipolar disorder; 7 (6.3%) with Unipolar Depression; 8 (7.2%) with Obsessive-compulsive Disorder and 8 (7.2%) with Personality Disorder. The most represented educational level among the subjects was primary high school (45%), then secondary school (38,7%) and graduation (5,4%). In addition, 97 participants (68.7%) lived with family; 13 (11,7%) came from other communities or protected residential structures and 3 (2,7 %) from prison facilities. According to the residential program, RHI patients were 78 (70.3%) while 33 (29.7%) subjects composed the RPA group. The mean age in these subgroups was 37,39 years ± 10,14 in RHI vs 53,51 years ± 7,54 in the RPA group.

The main socio-demographic variables of the total sample are summarized in Table 1.

**Table 1:** Socio-demographic variables

Socio-demographic variables (*N =* 111)	Prevalence % (mean ±SD)
Age	41.8 years ( ± 11.9)
Gender:	
Male	49.5%
Female	50.5%
Duration of illness	18 years ( ± 11.1)
Psychiatric diagnosis:	
Psychotic disorders	39.6%
Schizoaffective disorder	9%
Bipolar disorder	30.6%
Unipolar Depression	7.3%
Obsessive-compulsive Disorder	7.2%
Personality disorder	7.2%
Schooling:	
Primary high school	45%
Secondary school	38.7%
Graduation	5.4%
Been living:	
Family of origin	68.7%
Communities/Residential structures	11.7%
Prison facilities	2.7%
Residential program:	
High intensity	70.3%
Post-acute	29.7%

### Outcome Indicators

#### Hospitalizations

The *number* and *duration* of the inpatient treatment (days/year) were used as outcome indicators of hospitalizations in the 12 months preceding and following the rehabilitation program.

**Fig. 1:**
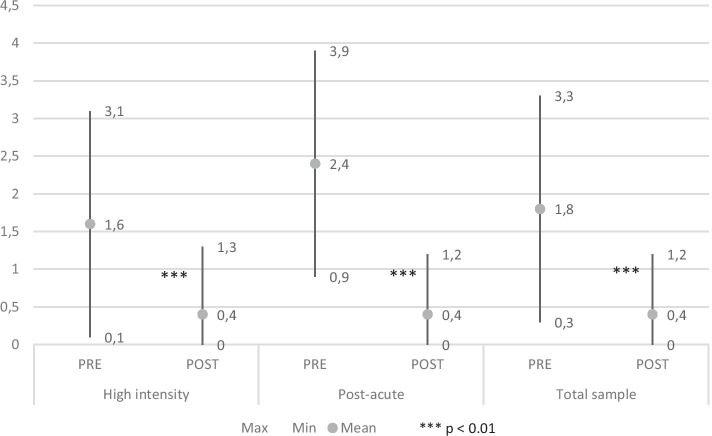
Number of hospitalizations

*Considering the total sample, the number of hospitalizations* before admission was 1.8±1.5 on average (min=0, max=8). After discharge the average value was 0.4±0.8 (min=0, max=4). In the RHI group the number of hospitalizations went from a mean value of 1.6±1.5 before vs 0.4±0.9 after; in the RPA group was 2.4±1.5 before admission vs 0.4±0.8 at the discharge. The reduction was statistically significant (*p* <0.001) in every subgroup (Fig. 1).

**Fig. 2:**
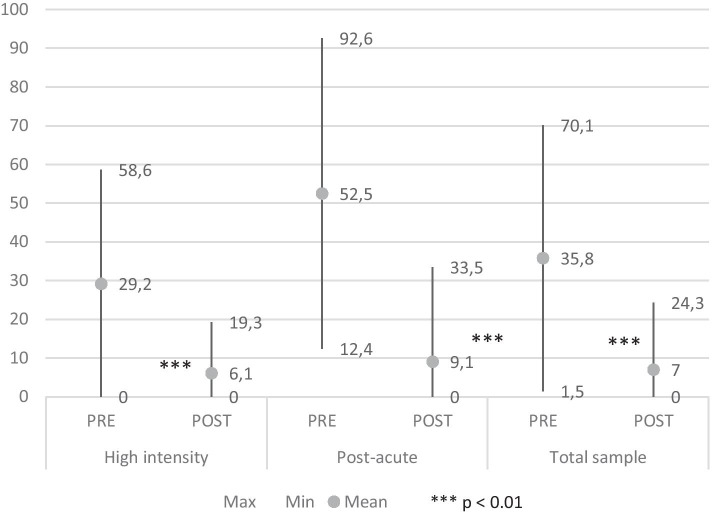
Duration of hospitalizations days/year

*The Duration of hospitalization* per year in the total sample was 35.8±34.3 days/year before the admission (min=0, max=193) compared to 7±17.3 days/year at the end of the program (min=0, max=100). In RHI subjects the average in the 12 months before admission was 29.2±29.4 and 6.1±13.2 during the following 12 months. Finally, in RPA subjects the mean duration was 52.5±40.1 days before vs 9.1±24.4 after the rehab program. The reduction was statistically very significant (*p* <0.001) in every subgroup (Fig. 2).

#### Continuity of Care

**Fig. 3:**
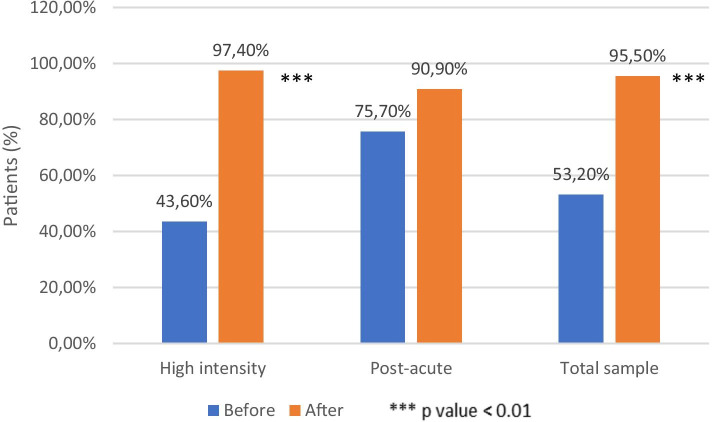
Continuity of care

The continuity of care is defined as at least one psychiatric examination in CPS (Psycho-Social Centre) every 90 days during a year-time period [42]. In the whole sample, during the year before the admission around half of the subjects (53.2%) were regularly visited in outpatients’ services, while during 12 months after discharge, almost all patients (95.5%) maintained regular contact with the CPS caregiver. The increase of continuity of care was statistically significant (*p* <0.001). Regarding RHI subgroup, continuity of care increased from 43.6% before admission to 97.4% after resignation, with a statistically significant difference (*p* <0.001). In post-acute patients as well, there was an increase in continuity of care (from 75.6%, to 90%) without a statistically significant difference (Fig. 3)

#### LAI Therapy Compliance

**Fig. 4:**
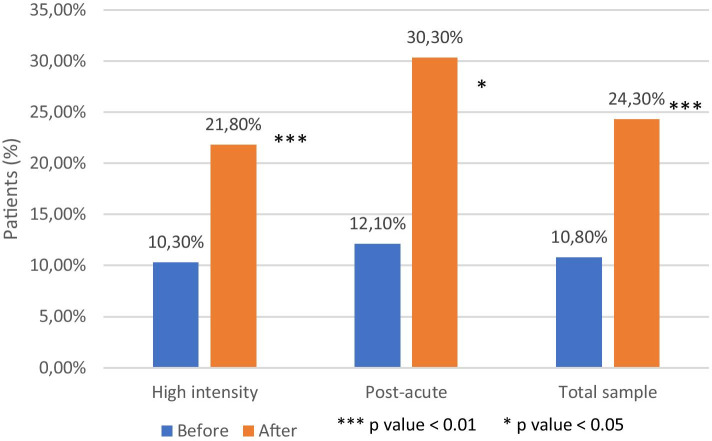
LAI therapy compliance

Compliance to therapy with long acting treatment in the total sample was 10.8% (10.3% high intensity and 12.1% post-acute) in the previous 12 months and 24.3% (21.8% high intensity; 30.3% post-acute) in the 12 months following discharge. The increase was statistically significant (*p* <0.001) in the general sample, and in both the RHI (*p* <0.01) and RPA (*p* <0.05) subgroup (Fig. 4)

#### Work and ALA

The collaboration with the Employment and Learning Agency (ALA - Agenzia Lavoro e Apprendimento) was also taken into consideration. ALA-Sacco is a second level Specialist Service, belonging to the Department of Mental Health of the University Hospital "Luigi Sacco”, promoted by Lombardia Region, which aims to help young psychiatric patients, with a good level of autonomy and functionality, in finding, approaching and maintaining a job. ALA also evaluates the selected patients from the CPS for working counseling and training. This service supports patients throughout the entire process from orientation, training, and work placement, acting as an intermediary with the different actors in the process (schools, companies, services, cooperatives).

**Fig. 5:**
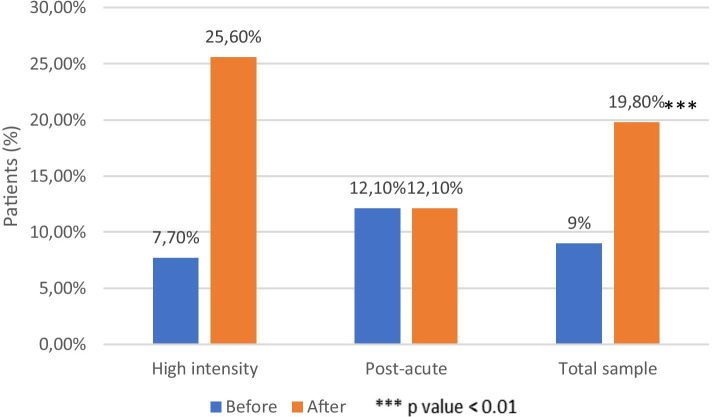
Stable employment in 12 months

The maintenance of a stable employment (at least 1 year) was considered as a positive outcome comparing the individual occupation 12 months before and after the residential program. Regarding the two subgroups, the high intensity group showed a significant improvement (from 7.7% to 25.6%, *p* <0.001) that was not confirmed in the post-acute group (12.1%, no change) (Fig. 5).

In the general sample, only one working patient out of 10 received ALA work support in the year before admission. At the end of the rehabilitation program, all worker patients (n=23) received ALA support intervention (20.7% of the total sample).

### Psychometric Analysis

#### BPRS

“Brief Psychiatric Rating Scale” is a scale composed of 24 items that can take a value from 1 to 7 depending on the presence, intensity and frequency of symptoms related to the psychopathological component of the subject. It includes positive and negative psychotic symptoms, suicidality, and also the prodromal symptoms of a relapse such as anxiety, depression, distractibility, emotional isolation, tension, restlessness, suspicion, somatic and hypochondriac concerns.

Considering the general sample, the BPRS values were 42,1 ± 9,8 at T0, 31,1 ± 8,7 at T1 and 34,9 ± 9,7 at T2. The value reduction from T0 to T1 was statistically significant (*p* <0,001) as well as the slight increase between T1 and T2 (*p* <0,001). The same pattern of statistical significance was observed in RHI (T0=41,3 ± 9,2; T1= 30,7 ± 7,3 ; T2= 34,3 ± 7,5; *p* <0,001) and in T0-T1 interval of RPA group (T0=42,4 ±10; T1= 31,3 ±9,3; *p* <0,001) while not in T1-T2 comparison (T2= 35,3± 10,8) of this group.

#### GAF

**“**Global Assessment of Functioning” is a global assessment scale developed following numerous reviews. It assesses the psychological, social and occupational functioning of the subject, regardless of the nature of the psychiatric disorder. It assigns numerical values from 1 (maximum severity of the disorder) to 100 (greater functioning in all areas), 0 identifies the lack of adequate information. For each area of operation, 10 levels of decreasing severity are defined.

**Fig. 6:**
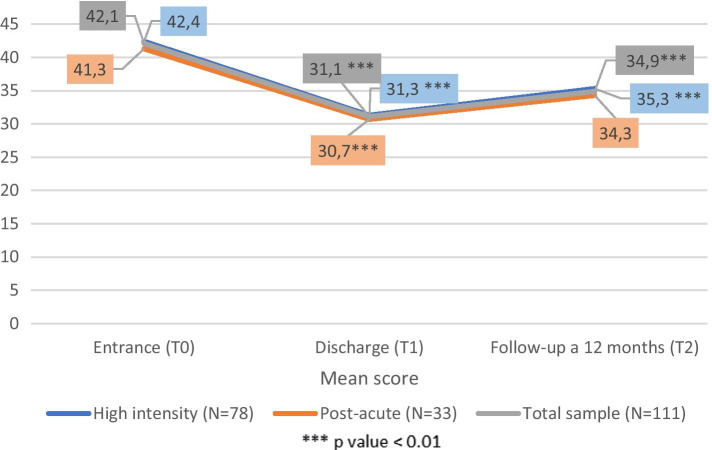
BPRS values

**Fig. 7:**
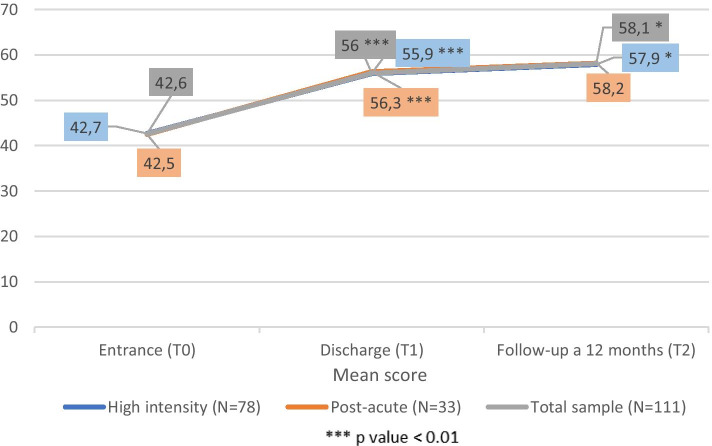
GAF values

GAF scores increased constantly in subsequent evaluations. Among the whole sample, GAF values rise from 42,67 ± 10,8 at T0 to 56 ± 11,2 at T1 (*p* <0,001) and then from T1 to T2 (58,1 ± 12,4; *p* <0,05). Among RHI and RPA subgroups a similar increasing trend was observed. Particular T0-T1 interval was statistically significant (*p* <0,001) in both the subgroups, while the T1-T2 interval was significant exclusively in RHI (*p* <0,05) (Figs. 6 and 7).

## Discussion

In the last few years, Recovery started to be considered by clinicians one of the main targets of treatment in mental health disorders. To reach this goal, a key role is represented by psychopharmacological compliance and community-based recovery services [31, 43, 44]. Several international studies have demonstrated the efficacy of those interventions, involving multiple treatment components and mental health specialists, individualized care plans and target outcomes [45, 46]. However Italian, as well as international data, did not suggest rehabilitation as an effective intervention and not include it in the usual clinical practice as underlined by several authors [31, 42, 47].

In order to obtain measurable results about the efficacy of rehabilitative community-based interventions, we suggested predictors of positive outcomes, according to available literature data and due to the simple feasibility. Different studies underlined the need of an outcomes evaluation model in community-rehabilitation programs, that take into account psychopathological, relational and social functioning [19–22].

The following weeks after discharge from the inpatient unit may represent a critical period especially in psychiatric patients. Facing and managing everyday life difficulties may increase isolation, anxiety, depression, suicidal risk, lack of compliance and continuity of care or integration in society in a sort of vicious circle [33, 48]. The present study suggests that RHI or RPA rehabilitation programs seem effective to reduce hospitalization in terms of episodes for year and inpatient duration in both the groups. Re-hospitalizations rate have always been considered as an indicator of quality of care since it reflects clinical stability [49]. All over the world, readmission rates have routinely been collected [50] and several strategies have been developed to reduce readmission rates [51]. The present study underlines the efficacy of recovery-approach treatment showing a statistically significant reduction in hospitalization per year in both the subgroups. (RHI and RPA patients). This result is consistent with previous data and is correlated with other outcome indicators as continuity of care. Moreover Lien et al. reported that a long outpatients follow-up might reduce readmission rates [52].

The continuity of care during the year before the rehabilitation interventions was lacking (just over half of the subjects), while it has greatly improved during the 12 months following discharge, reaching almost the totality of the sample (95% of general sample). Focusing on the RHI group, this outcome has shown a statistically significant improvement, from 43.6%, to 97.4% at the end of the program. This data may be explained partially by the fact that the recovery-model has helped patients to develop more insight about their disease [33, 53]. In fact, psychiatric rehabilitation generates individual satisfaction not only by realizing the rehabilitative goals, but also improving subjective well-being and insight of the disease [54] leading to a better compliance. Moreover, no adherence to medication is one of the most serious issues in long-term treatment of patients with serious mental illness [55] and discontinuity of psychopharmacological treatments leads to an increase of relapses and, doing so, hospitalization [56]. The statistically significant increase of LAI therapy in RHI and RPA subgroups, comparing 12 months before and after the rehabilitative intervention, underline that community recovery-based treatment might help patients to understand the importance of consistent pharmacological treatments in order to focus on their own life goals avoiding relapses and subsequent hospitalization [57]. Another possible explanation for this result may be the establishment of a trustful therapeutic relationship between patients and healthcare professionals, leading to better compliance and help seeking in case of emerging psychiatric symptoms.

Since work functioning is one of the main aspect of effective rehabilitative intervention, several studies shown that psychiatric patients are at higher risk of losing job and the employment rate was lower than general population, ranging from 10% to 25% [35, 37]. Our community programs aimed to empower patients to develop individual skills and to increase the self-esteem in order to improve work skills and employment maintenance [58]. According to this, there was an improvement in occupations in the year after community rehabilitation in RHI. Conversely, in the RPA group there wasn’t any significant change likely because of the RPA duration (only three months), not long enough to develop and maintain the necessary work skills. Moreover, RPA patients’ age limited the involvement in ALA services and similar working supporting facilities [59]. The increase of working patients in the RHI group is a significant result since work improves individual functioning, self-esteem and quality of life [60, 61].

Nevertheless, the data about indicators of outcome in recovery-based communities are little available. Even if previous studies have evaluated the efficacy of mental health rehabilitation [10], only a few evaluate follow up after discharge [10]. Hence, there is no evidence about the improvements in maintenance over time [31]. In the present study we analyzed all the outcome indicators after twelve months showing improvements of recovery-based rehabilitative intervention and their maintenance over at least 12 months. To strengthen this evidence GAF and BPRS were performed in T0, T1 and T2. The efficacy of rehabilitative intervention is underlined by the significant reduction of BPRS values and increase of GAF scores. Moreover, comparing T1 and T2 values, GAF showed a further increase in individual functioning while T2 BPRS score was slightly higher than T1 ones, but significantly lower than T0. The slight increase in T2 BPRS scores may represent a signal of a slow and progressive relapse or a subjective reaction to the discharge from communitarian environment and the return in their own ordinary and stressful daily life. These last evidences may confirm quantitatively the improvement and maintenance over time of rehab clinical results in the general sample as well as in both RHI and RPA groups.

### Strength and Limitations

To our knowledge, this is the first Italian study providing evidence of the efficacy of the recovery-model community evaluating outcome indicators in patients, 12 months before and after a community rehabilitation treatment. This observational retrospective study analyzed not only the multiple psychopathological, relational and social factors that affected different areas of community functioning, but also clinical outcomes variables (continuity of care, working condition, LAI compliance and re-hospitalizations). In order to reach this goal, we applied and developed standardized sets of outcomes used to evaluate community recovery-centered efficacy. Another strength of this study may be represented by the easy availability of outcome data both during the rehabilitative intervention and follow up time thanks to regional SISM.

However, there are several limitations in this study. First, this study only recruited patients from one public Italian community, excluding the ones with the rehabilitation project still in progress. This may cause an under-representation of psychiatric patients in the local territory. Second, we considered psychiatric patients with mixed diagnosis and in different stages of their disease. This might represent a confounding element especially in terms of outcomes. The authors have decided to maintain this unique sample, so the results might be better representative of the psychiatric population with different mental disorders. Third, although this study has identified key variables that could be predictors of a positive outcome in a community recovery-centered, researchers should conduct more studies to create a standardized outcomes model in different settings. The validity of this study is also limited by the broad range of rehabilitative interventions provided during permanence in CRA and the different timing of outcome evaluation due to individual rehabilitation programs. Lastly, a longer follow up should be performed since the lifetime course of psychiatric disease and since the impact of patient’s “ordinary” environment and relations upon the improvements gained during community admission.

## Conclusion

This study identified variables (continuity of care, working condition, LAI compliance and re-hospitalizations) that might be easily collected in clinical practice and representative of goals in psychiatric rehabilitation in order to evaluate and compare rehabilitative program since the actual lacking of uniform and validated evaluative model in this field as underlined by several studies [62, 63]. In this perspective, multiple outcome comparisons have been performed considering 12 months before and after the rehabilitative interventions, to confirm over time the achievement reached during the rehabilitative program. Our results suggest that community-based rehabilitation program was effective in improving individual functioning and clinical stability through the recovery process as confirmed by positive indicators selected: consistent continuity of care, in terms of psychiatric outpatient evaluation and LAI compliance; reduction in hospitalizations; job placement and maintenance. The positive results of this study suggest the feasibility of the presented outcome indicator model, in terms of evaluation of rehab intervention efficacy and possibility of clinical applicability. Besides numerous rehabilitation interventions papers, further studies are required to rigorously define an outcomes evaluation model of community-rehabilitation recovery-centered. This study also highlights the importance of a clinical and rehabilitative intervention characterized by a systematic data collection. Any type of rehabilitative intervention, conducted on psychiatric patients, both in residential and other contexts, may be inadequate if it is not possible to assess its concrete effectiveness, in the short and long term, through a systematic analysis of the outcomes of the intervention itself. These are necessary to ensure the development of a recovery-oriented rehabilitation model and therefore its reproducibility.

## Data Availability

The datasets generated during and/or analysed during the current study are available from the corresponding author on reasonable request.
